# ASSOCIATION BETWEEN TIME SPENT OUTSIDE THE HOME AND EXECUTIVE FUNCTION IN PATIENTS WITH PAST TRAUMATIC BRAIN INJURY

**DOI:** 10.1093/geroni/igad104.3193

**Published:** 2023-12-21

**Authors:** Emily Richards, Patrick Donahue, Kyle Moored, Dana Eldreth, Breanna Crane, Michelle Carlson

**Affiliations:** Johns Hopkins University, Baltimore, Maryland, United States; Johns Hopkins University, Baltimore, Maryland, United States; Johns Hopkins Bloomberg School of Public Health, Baltimore, Maryland, United States; University of Illinois Urbana Champaign, Urbana, Illinois, United States; Johns Hopkins Bloomberg School of Public Health, Baltimore, Maryland, United States; Johns Hopkins Bloomberg School of Public Health, Baltimore, Maryland, United States

## Abstract

Time spent out of the home and in the surrounding neighborhood has numerous physical health benefits, but the cognitive benefits are less clear. Evidence suggests that executive functions are important to navigating activity outside the home. This study examined whether time spent outside in participants’ immediate neighborhoods was more strongly associated with executive function than memory. Participants were adults with mild cognitive impairment and history of traumatic brain injury (TBI) enrolled in an immersive computer game intervention (N=11; 63.6% male; mean=59.0 years, range: 42-75). At baseline, participants completed the Life Space Questionnaire (LSQ) and cognitive tests of Stroop (executive function), Rey Auditory Verbal Learning Test (RAVLT) (memory), and Pattern Comparison Test (PCT) (processing speed). Cognitive tests were performed on an iPad to precisely measure reaction time. Pearson correlations were used to examine the association between the LSQ and cognitive measures. Although time spent in the neighborhood was not statistically associated with performance on cognitive measures, the magnitude of the correlations was noteworthy. After adjusting for age and education, days spent in the neighborhood were more strongly associated with better executive function (Stroop) (r=-0.58, p=0.10) and processing speed (PCT) (r=-0.27, p=0.49). Additionally, neighborhood exposure explained a larger proportion of the variance in Stroop and PCT rather than in RAVLT. In patients with past TBI, time spent outside of the home in the neighborhood may be associated with better executive functions through engagement with one’s environment. We will further describe objective measures of time spent outside the home using GPS.

